# Holistic understanding of the response of grapevines to foliar application of seaweed extracts

**DOI:** 10.3389/fpls.2023.1119854

**Published:** 2023-02-24

**Authors:** Iratxe Zarraonaindia, Enrico Cretazzo, Amaia Mena-Petite, Ana M. Díez-Navajas, Usue Pérez-López, Maite Lacuesta, Eva Pilar Pérez-Álvarez, Belén Puertas, Catalina Fernandez-Diaz, Nadia Bertazzon, Emma Cantos-Villar

**Affiliations:** ^1^ Department of Genetics, Physical Anthropology and Animal Physiology, Faculty of Science and Technology, University of the Basque Country Universidad del País Vasco/Euskal Herriko Unibertsitatea (UPV/EHU), Leioa (Bizkaia), Spain; ^2^ IKERBASQUE, Basque Foundation for Science, Bilbao, Spain; ^3^ Instituto de Investigación y Formación Agraria y Pesquera (IFAPA) Rancho de la Merced, Consejería de Agricultura, Pesca, Agua y Desarrollo Rural, Junta de Andalucía, Cádiz, Spain; ^4^ Department of Plant Biology and Ecology, Faculty of Pharmacy, University of the Basque Country Universidad del País Vasco/Euskal Herriko Unibertsitatea (UPV/EHU), Vitoria-Gasteiz (Araba), Spain; ^5^ Department of Plant Production and Protection, Instituto Vasco de Investigación y Desarrollo (NEIKER)-Basque Institute of Agricultural Research and Development, Basque Research and Technology Alliance (BRTA), Arkaute (Araba), Spain; ^6^ Department of Plant Biology and Ecology, Faculty of Science and Technology, University of the Basque Country Universidad del País Vasco/Euskal Herriko Unibertsitatea (UPV/EHU), Leioa (Bizkaia), Spain; ^7^ VIENAP Group, Instituto Vasco de Investigación y Desarrollo (ICVV), Carretera de Burgos, Logroño, Spain; ^8^ Instituto de Investigación y Formación Agraria y Pesquera (IFAPA) El Toruño, Consejería de Agricultura, Pesca, Agua y Desarrollo Rural, Junta de Andalucía, Cádiz, Spain; ^9^ The Council for Agricultural Research and Economics (CREA), Research Centre for Viticulture and Enology, Conegliano, Italy

**Keywords:** *Ulva ohnoi*, *Rugulopteryx okamurae*, biostimulation, *PR protein* genes, stilbenes, jasmonic acid, superoxide dismutase, microbiota

## Abstract

Viticulture is highly dependent on phytochemicals to maintain good vineyard health. However, to reduce their accumulation in the environment, green regulations are driving the development of eco-friendly strategies. In this respect, seaweeds have proven to be one of the marine resources with the highest potential as plant protective agents, representing an environmentally-friendly alternative approach for sustainable wine production. The current work follows an interdisciplinary framework to evaluate the capacity of *Ulva ohnoi* and *Rugulopteryx okamurae* seaweeds to induce defense mechanisms in grapevine plants. To our knowledge, this is the first study to evaluate *Rugulopteryx okamurae* as a biostimulator . This macroalgae is relevant since it is an invasive species on the Atlantic and Mediterranean coast causing incalculable economic and environmental burdens. Four extracts (UL1, UL2, RU1 and RU2 developed from *Ulva* and *Rugulopteryx*, respectively) were foliar applied to Tempranillo plants cultivated under greenhouse conditions. UL1 and RU2 stood out for their capacity to induce defense genes, such as a *PR10, PAL, STS48* and *GST1*, mainly 24 hours after the first application. The increased expression level of these genes agreed with i) an increase in *trans*-piceid and *trans*-resveratrol content, mainly in the RU2 treated leaves, and, ii) an increase in jasmonic acid and decrease in salicylic acid. Moreover, an induction of the activity of the antioxidant enzymes was observed at the end of the experiment, with an increase in superoxide dismutase and catalase in the RU2-treated leaves in particular. Interestingly, while foliar fungal diversity was not influenced by the treatments, alga extract amendment modified fungal composition, RU2 application enriching the content of various groups known for their biocontrol activity. Overall, the results evidenced the capacity of *Rugulopteryx okamurae* for grapevine biostimulation, inducing the activation of several secondary metabolite pathways and promoting the abundance of beneficial microbiota involved in grapevine protection. While further studies are needed to unravel the bioactive compound(s) involved, including conducting field experiments etc., the current findings are the first steps towards the inclusion of *Rugulopteryx okamurae* in a circular scheme that would reduce its accumulation on the coast and benefit the viticulture sector at the same time.

## Introduction

Seaweeds are macroalgae with high nutritional, nutraceutical and medicinal properties. Their use as fertilizers in agriculture has evolved recently as they are beneficial to crops in several ways. They stimulate seed germination, enhance plant health and growth through shoot and root elongation, improve water and nutrient uptake by the plant, promote frost and saline resistance, and remediate pollutants from contaminated soils (Beckers and Spoel, 2006). In addition, polysaccharides (e.g. ulvan, laminarin) or lipids (e.g. terpenes, fucoxantines) extracted from seaweed have proven to induce resistance towards phytopathogenic organisms by stimulating the natural defenses of plants (Nabti et al., 2017). Plant biostimulants are “fertilizing products able to stimulate plant nutrition processes independently of the products’ nutrient content” according to the recent European Union regulation (https://eur-lex.europa.eu/legal-content/EN/TXT/PDF/?uri=CELEX:32019R1009&from=EN). Based on the European Commission report, seaweed extracts, including both macroalgae and microalgae, make up to 40% of the total biostimulant market (European Commission 2009) (https://eur-lex.europa.eu/resource.html?uri=cellar:5aa49d31-ec29-11e5-8a81-01aa75ed71a1.0001.02/DOC_3&format=PDF). The exposure of plants to seaweed- derived products induces the transduction of various plant signaling pathways, which leads to the synthesis and synchronized accumulation of defensive molecules, some of which play a structural role, while others exert a direct antimicrobial function (Stadnik and de Freitas, 2014; Bouissil et al., 2019). Therefore, seaweed application is considered one of the most promising and sustainable alternative strategies for protecting crops against biotic and abiotic stressors.

The edible green seaweed of the genus *Ulva* belongs to the Ulvaceae family of green macroalgae and is one of the most common shallow-water seaweeds found around the world. *Ulva* species have been shown to contain several direct antifungal compounds, such as proteins, fatty acids and aromatic compounds, many of which are suggested to have direct antifungal properties (Shomron et al., 2022). Ulvan is the main water-soluble, sulfur-containing polysaccharide in *Ulva*. Ulvan extract has been shown to activate plants’ jasmonic acid signaling pathway, involved in the induction of defense mechanisms (Beckers and Spoel, 2006). Moreover, ulvan protected grapevines from p owdery mildew disease and *Botrytis cinerea* pathogen (Jaulneau et al., 2011; Shomron et al., 2022). In fact, this polysaccharide capacity to elicit plant immune responses has been shown to be a promising way to reduce agricultural reliance on traditional pesticide treatments (Kidgell et al., 2019).

*Rugulopteryx okamurae* is a brown alga belonging to the Dictyotaceae family, originally from the coasts of the warm and temperate northwestern Pacific Ocean. It was introduced to the Mediterranean through the Strait of Gibraltar, where it has found a highly favorable environment. From 2015 to 2020, it exhibited extensive northerly and southerly geographical expansion, along both the Atlantic and the Mediterranean coasts, causing a considerable negative environmental impact. In 2020 it was ranked among the most invasive non-indigenous species in the Mediterranean Sea and included in the Spanish Catalog of Invasive Exotic Species, since it represents one of the main threats to the biodiversity in the Mediterranean (García-Gómez et al., 2020; Santana et al., 2022). Five thousand tons of this Asian alga were removed from the beaches of Ceuta (North of Africa, Spain) in 2015, and 400 tons from the beaches of Tarifa (Andalucía, Spain) in July 2020 alone (Personal communication Dr. Hachero-Cruzado). Thus, the valorization of the biomass of this macroalgae can provide an incentive for its withdrawal and control. Regarding its biochemical composition and bioactivity, little is yet known. Recently, Cuevas et al. (2021) reported the anti-inflammatory capacity of several terpenoids derived from this alga. Similar to other species classified within brown seaweeds, alginates and fucoidans are expected to be present in *Rugulopteryx okamurae*. Both polysaccharides, extracted from the most widely studied brown algae, *Ascophyllum nodosum* and *Laminaria digidata*, have already proven their biostimulant activity (Bittkau et al., 2020; Samuels et al., 2022). However, to our knowledge, no studies have analysed *Rugulopteryx okamurae* fertilizer or its biostimulant properties in agriculture.

Viticulture is a sector with great socioeconomic importance worldwide, Europe having the largest cultivated area, the top wine producing countries being Italy, France and Spain (https://www.oiv.int/what-we-do/statistics). However, grapevine functioning, development and production face growing pressures associated with abiotic (drought, salt, mineral nutrition disturbances, light and temperature) and biotic (wounding, pathogens, and herbivores) stressors, all of which contribute to the overuse of chemical fertilizers and synthetic pesticides (Samuels et al., 2022). The use of biostimulants derived from algae applied as foliar sprays onto grapevines could be a suitable strategy to promote sustainability in viticulture. These substances have been shown to affect vine growth parameters (Salvi et al., 2019; Monteiro et al., 2022; Samuels et al., 2022), secondary metabolites such as polyphenolic compounds (Krzyzaniak et al., 2018), antioxidant enzymes and hormones (Salvi et al., 2019; Monteiro et al., 2022; Samuels et al., 2022), and increase the abundance of particularly beneficial microbial groups (Perazzolli et al., 2020). All in all, this could enhance the protection of grapevines against stressors and thereby reduce the accumulation of chemical compounds in soil and vines.

Seaweed extracts, or purified molecules from them, are able to induce defense reactions through a cascade of signaling events previously described (Delaunois et al., 2014; Bodin et al., 2020). However, despite the great interest in developing and testing new seaweed extracts as biostimulants, few well-characterized products with reliable performance are available on the market, with those that are deriving mainly from *Ascophyllum nodosum* (Jindo et al., 2022). Therefore, the objective of the current work is to provide an comprehensive overview of the vine response to seaweed application by studying the biostimulant efficiency of *Ulva ohnoi* and *Rugulopteryx okamurae* extracts through different layers (genetics, plant physiology, secondary metabolites and microbiology) to provide insights into their activity. With this aim in mind, four crude extracts (two per macroalgae) were developed and biochemically characterized. Then, the four crude seaweed extracts w ere foliar applied to vines of a Tempranillo (*Vitis vinifera* L.) cultivar under greenhouse-controlled conditions. The immune and physiological response of the vines after one or two foliar applications was addressed by targeting the expression of immune-related genes, phenolic compounds, phytohormone levels and oxidative-related enzymes. The implications for vine development were also addressed (e.g. plant growth and photosynthetic capacity). In addition, the impact of seaweed on leaf fungal diversity and structure was evaluated through Next Generation Sequencing. Reducing the dependence of the viticultural sector on chemical inputs while contributing to the blue bioeconomy will help in achieving the objectives of the European Green Deal, which seeks to transition to a green, circular and carbon neutral EU.

## Material and methods

### Algae extracts elaboration and biochemical composition

The green macroalgae *Ulva ohnoi* was provided by “La Huerta Marina” (Huelva, Spain, 7° 09´41.8′′W, 37°15´20.9′′N). The brown *Rugulopteryx okamurae* macroalgae was collected in the area near Algeciras (Cadiz, Spain, 5°25′34.75′′W, 36°4′37.56′′N). After harvesting, the seaweed biomass was rinsed with tap water, freeze-dried (Cryodos, Telstar, Spain) and milled to a fine powder and kept in dry conditions until the preparation of the extracts.

Two different extracts were generated with each alga, hereafter UL1 and UL2 for *Ulva ohnoi*, and RU1 and RU2 for *Rugulopteryx okamurae*. UL1 was provided by “La Huerta Marina” (ECOALGA^®^, Huelva, Spain) and was formulated without the addition of conserving agents to prevent any possible interference. UL2 extract was generated following the protocol of Coste et al. (2015) with modifications. Briefly, 50 g of freeze-dried algae were combined with 500 mL of milliQ water (70 °C, 2 hours, and shaking) twice. The aqueous solution was frozen and freeze-dried. RU1 extraction started from 50 g of algae with 500 mL of milliQ water (70 °C, 2 hours, and shaking). The residue was re-extracted with a water:ethanol (20:80) solution. The two liquid phases were combined, and after lyophilization, a crude extract of *Rugulopteryx* was obtained. RU2 was created following the same protocol as for UL2.

The composition of each algae extract was characterized. Their ash content was measured by heating the sample overnight in a furnace at 525 °C, and the content was determined gravimetrically. CNHS content was determined by the Institute of Marine Sciences of Andalusia (ICMAN-CSIC) using a CHNS elemental analyzer (Thermo Fisher Scientific, USA). The soluble proteins present in the extracts were calculated from the nitrogen content. Lipids were measured using methods described in (Folch et al., 1957). The total carbohydrate content was determined using the phenol–sulfuric acid method (DuBois et al., 1956). In addition, *L*-fucose was determined following a method developed by Dische and Shettles (1948) and modified according to January et al. (2019) for microplates. Uronic acid was evaluated by a method first developed by Dische (1947) and later optimized by Cesaretti (2003) using glucuronic acid as a standard. The content of sulfates in the different algae extracts was determined following the protocol described in Torres et al. (2021).

Macroelements (Ca, K, Mg, P, Na), microelements (Fe, Mn, Cr, Mo, Cu, Zn, and Se) and heavy metals (Cd, Hg, Pb, and As) were simultaneously analyzed at the ICMAN-CSIC by Inductively Coupled Plasma Optical Emission spectrometry (ICP-OES) using a Perkin–Elmer Optima 4300 DV spectrometer (Shelton, CT, USA).

### Plant material, greenhouse treatments and sampling

Grapevine plants (*Vitis vinifera* L. cv. Tempranillo grafted on R-110 rootstock) provided by a commercial nursery (Vitis Navarra, Navarra, Spain) were grown in a level 2 biosafety greenhouse. The grapevines were placed in 5 L pots in an enriched nutrient substrate containing organic matter 90 %, Sphagnum peat (160 g/L), calcium carbonate (7 g/L), NPK fertilizer (1.5 g/L) and trace elements (PotgrondH, Klasmann-Deilmann GmbH, Germany). The plants were grown for 2 months with a 16h day/8h night photoperiod, at an average room temperature of 18 °C, and were irrigated to field capacity when necessary.

**Figure 1:**
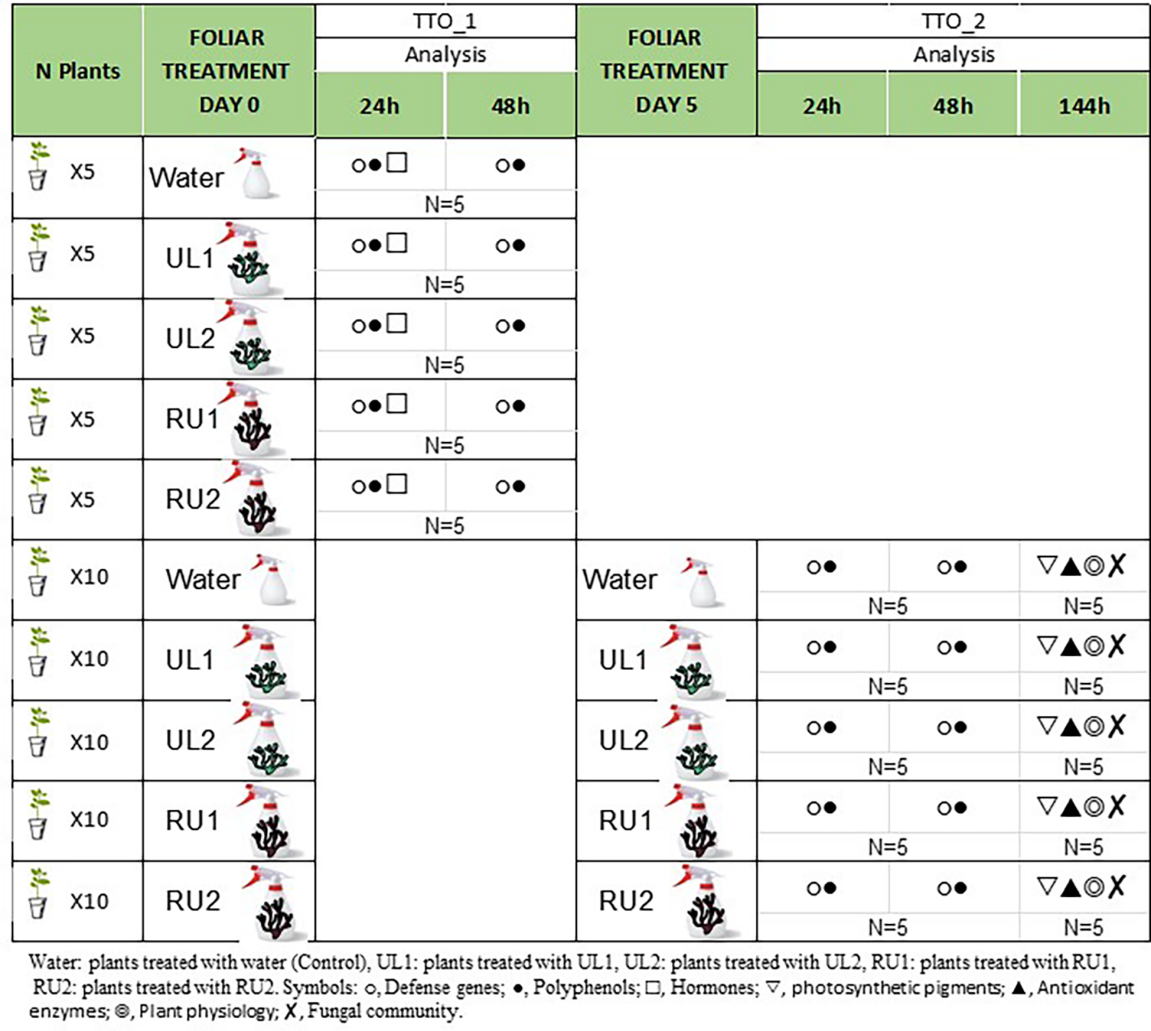
Experimental design.

Plants with 10-12 leaves were used for the experiment (75 plants). An initial foliar treatment of water (CT), UL1, UL2, RU1 or RU2 was applied to the grapevines (**Figure 1**) at a concentration of 6 g/L. All the treatments contained 0.1 % of retenol^®^ (Daymsa, Zaragoza, Spain) as an adjuvant. A batch of the plants received a second application six days after the first one at the same dose (6 g/L). The plants that received either one or two applications per treatment (N= 5 plants, per treatment and application) were used to evaluate gene expression and polyphenols 24 and 48 hours after each application (referred as TTO_1, 24h and TTO_2, 48h, **Figure 1**). The evaluation of hormones was performed at TTO_1, 24h. The 4^th^ and 5^th^ leaf starting from the apex was collected at each time point, respectively. Additional plants receiving two applications per treatment were evaluated at the end of the experiment (12 days after first application, TTO_2, 144h) for oxidative enzyme determination (5^th^ leaf), microbiota analysis (4^th^-8^th^ leaves) and physiological parameters including plant height, root weight and photosynthetic pigments. The leaf samples collected were frozen in liquid nitrogen and stored at -80 °C until analysis.

### Gene expression analysis

Expression changes of fifteen defense and stress-related genes (**Table 1**) were quantitatively determined by real-time PCR assay (RT-qPCR). More specifically, this evaluated the following: the expression of six genes encoding pathogenesis-related-proteins (*PR*) (Beta-1,3-glucanase (PR2), Chitin binding Chitinases type I, II (PR4), Thaumatin-like/Osmotin (PR5), Proteinase inhibitor (PR6), Chitinase type III (PR8),Ribonuclease-like (PR10)); four genes from the phenylpropanoid pathway (phenylalanine ammonia lyase (PAL), and stilbene synthase group A (STS1), B (STS16) and C (STS48); three genes involved in flavonol and anthocyanin biosynthesis (chalcone synthase1 (CHS1), dihydroflavonol reductase (DFR), leucoanthocyanidin dioxygenase (LDOX); a gene involved in redox status regulation (glutathione-S-transferase (GST1); and a transcription factor gene (WRKY1). Reactions were carried out in a CFX Connect Real-Time PCR system (Bio-Rad Laboratories, Hercules, CA, USA). Each assay was done in a final volume of 10 μL, consisting of 5 μL SsoAdvanced Universal SYBR Green Supermix (Bio-Rad Laboratories, Hercules, CA, USA), 2 μL diluted cDNA template, 400 nM of each primer and nuclease free water. The thermal cycling conditions were as follows: an initial denaturation phase at 95 °C for 3 mins, followed by 39 cycles at 95 °C for 15 s and 60 °C for 30 s. According to the melting temperature, for some primer pairs the 30 s annealing-extension step was split into two sub-steps at 55 °C (10 s) and 60 °C (25 s), respectively. At the end of each RT-qPCR run, the specificity of the primer annealing was confirmed by melting curves.

**Table 1:** Defense and stress-related genes studied.

Pathway	Gene name	Code	Primers from
PR protein	PR2 (Beta-1,3-glucanase)	PR2	Dufour et al., 2016
	PR4 (Chitin binding Chitinases type I, II)	PR4	Dufour et al., 2016
	PR5 (Thaumatin-like/Osmotin)	PR5	Dufour et al., 2016
	PR6 (Proteinase inhibitor)	PR6	Bertazzon et al., 2019
	PR8 (Chitinase type III)	PR8	Dufour et al., 2016
	PR10 (Ribonuclease-like)	PR10	Dufour et al., 2016
Phenylpropanoid metabolism	phenylalanine ammonia lyase	PAL	Repetto et al., 2012
	stilbene synthase (group A)	STS1	Sparvoli et al., 1994
	stilbene synthase (group B)	STS16	Vannozzi et al., 2012
	stilbene synthase (group C)	STS48	Vannozzi et al., 2012
Flavonols and anthocyanins biosynthesis	chalcone synthase1	CHS1	Gutha et al., 2010
	dihydroflavonol reductase	DFR	Gutha et al., 2010
	leucoanthocyanidin dioxygenase	LDOX	Gutha et al., 2010
Redox status regulation	glutathione-S-transferase	GST1	Dufour et al., 2016
	WRKY1 transcription factor	WRKY1	Repetto et al., 2012
Reference	cytochrome oxidase	COX	Bertazzon et al., 2012
	pyruvate decarboxylase	PDC	Bertazzon et al., 2012
	glyceraldehyde-3-phosphate dehydrogenase	GAPDH	Bertazzon et al., 2012

Two technical replicates were run for each of the independent biological replicates, and the geometric mean of the expression ratios of three reference genes (**Table 1**) was used to normalize transcript expression levels. The comparative Ct (2−ΔΔCt) method was used to calculate the transcript expression levels, as all the genes showed similar amplification efficiencies (between 90 and 100 %). The expression profile of defense marker genes was determined by comparing treated and untreated plants at each time point. A heatmap to visualize under- and overexpression of genes was generated using the Heatmapper web server (http://www.heatmapper.ca/).

### Phenolic compounds

Polyphenols were extracted from the leaves following the method described by Krzyzaniak et al. (2018). Briefly, 50 mg of freeze-dried powdered leaves was extracted with 1 mL of methanol for 15 min at 22 °C in an ultrasonic bath. The supernatant was recovered and the process was repeated four times. The supernatants were evaporated, redissolved in methanol:water (1:5), and then filtered through a 0.22 µm filter (PTFE Teknokroma, Barcelona, Spain). The samples (20 μL) were analyzed using a Waters HPLC system (Milford, MA, USA) equipped with a model 1525 pump, W2707 injector, and Waters 2996 photodiode detector and a Mediterranean Sea C18 column (Tecknokroma, Barcelona, Spain) (RP-18, 25 × 0.46 cm; 5 μm particle size) with a precolumn of the same material. Hydroxycinnamic acids were quantified at 320 nm as caffeic acid, while stilbenes were quantified at 306 nm as resveratrol. Concentrations were expressed in mg/L. A one-way analysis of variance (ANOVA) of the mean values was used to test for differences between time points and treatments. Significant results (p ≤ 0.05) were then evaluated with Tukey’s test using Statistix version 9.0 (Analytical Software, Tallahassee, FL, USA).

### Endogenous plant hormones

The main classes of plant hormones, namely cytokinins [*trans*-zeatin (t-Z), zeatin riboside (ZR) and isopentenyladenine (iP)], indole acetic acid (IAA), abscisic acid (ABA), jasmonic acid (JA) and salicylic acid (SA), were extracted and analyzed at the first sampling point (TTO_1, 24h, **Figure 1**) as described previously in Albacete et al. (2008) with some modifications. Lyophilized plant material (50 mg) was homogenized in liquid nitrogen and incubated in 1 mL of cold (-20 °C) extraction mixture of methanol:water (80:20) for 30 min at 4 °C. Solids were separated by centrifugation (20,000 g, 15 min at 4 °C) and re-extracted for another 30 min at 4 °C with 1 mL of extraction solution. Pooled supernatants were passed through Sep-Pak Plus C_18_ cartridges (previously conditioned with 3 mL of extraction buffer) to remove interfering lipids and some plant pigments. The supernatant was collected and evaporated under vacuum at 40 °C. The residue was dissolved in 1 ml methanol:water (20:80) solution using an ultrasonic bath. The dissolved samples were filtered through 13 mm diameter Millex filters with a 0.22 μm pore size nylon membrane (Millipore, Bedford, MA) and placed into opaque microcentrifuge tubes. Ten microliters of filtered extract were injected into an ultra-high performance liquid chromatography (UHPLC) system coupled with mass spectrometry (MS) consisting of an Accela Series U-HPLC (ThermoFisher Scientific, Waltham, MA) coupled to an Exactive mass spectrometer (ThermoFisher Scientific, Waltham, MA) using a heated electrospray ionization (HESI) interface. Mass spectra were obtained using the Xcalibur software version 2.2 (ThermoFisher Scientific, Waltham, MA). To quantify plant hormones, calibration curves were constructed for each analyzed component (0, 1, 10, 50 and 100 μg/L).

SPSS 28.0 (IBM Corp. Armonk, NY: IBM Corp, USA) was used for the statistical analysis. A one-way analysis of variance (ANOVA) of mean values was used to test for differences between treatments. Tukey’s test was used to compare all the samples tested when significant differences were observed by ANOVA (p ≤ 0.05).

### Antioxidant enzymes

The activity of enzymes including superoxide dismutase (SOD, EC 1.15.1.1), ascorbate peroxidase (APX, EC 1.11.1.11), catalase (CAT, EC 1.11.1.6) and glutathione reductase (GR, EC 1.6.4.2) was determined as described in Pérez-López et al. (2009). Briefly, grinded leaf tissue (0.15 g fresh weight) was extracted in a specific buffer (3 mL) for each enzyme extraction. The homogenates were centrifuged at 16.100 g for 25 min. SOD activity was assayed by the ferricytochrome-c reduction spectrophotometric test, using xanthine/xanthine oxidase as the source of superoxide radicals. One unit of SOD was defined as the amount of enzyme that inhibited the rate of ferricytochrome-c reduction by 50 %. CAT activity was measured spectrophotometrically, by monitoring the disappearance of H_2_O_2_ at 240 nm. To measure GR activity, the GSSG (oxidized glutathione) dependent oxidation of NADPH was monitored by the decrease in absorbance at 340 nm at 25 °C. APX activity was assayed by measuring the oxidation of ASA (ascorbate) at 290 nm.

SPSS 28.0 (IBM Corp. Armonk, NY: IBM Corp, USA) was used for the statistical analysis. A one-way analysis of variance (ANOVA) of mean values was used to test for differences between treatments. Tukey’s test was used to compare all the samples tested when significant differences were observed by ANOVA (p ≤ 0.05).

### Vine development and photosynthetic pigments

Plant growth parameters were evaluated, determining the number of leaves per plant and the stem height (cm) at the end of the experiment (TTO_2, 144h, **Figure 1**). In addition, the root system was weighed (fresh weight, FW) and then oven dried at 65 °C for at least 72 hours (dry weight, DW) to calculate the FW/DW ratio.

Measurements of the photochemical efficiency of photosystem II (*φ_PSII_*) and the leaf chlorophyll level (SPAD index) were performed *in situ* by a FluorPen FP 100 fluorometer (Photon Systems Instruments, Brno, Czech Republic) and a Konica-Minolta SPAD-502 Plus, respectively, in accordance with the authors Pérez-López et al. (2012) and Yuan et al. (2016).

Chlorophyll a, chlorophyll b, and carotenoids were determined according to Pérez-López et al. (2015). Briefly, 25 to 50 mg of ground powder were extracted with 1,5 mL dimethyl sulfoxide for 2 hours at 80 °C. Absorbances were determined at 750, 665, 649, and 480 nm.

Statistix version 9.0 (Analytical Software, Tallahassee, FL, USA and SPSS 28.0, IBM Corp.) was used for the statistical analysis. Tukey’s test was used to compare all the samples tested when significant differences were observed by ANOVA (p ≤ 0.05).

### Fungal community diversity and structure

A total of 180 mg of the ground homogenized leaf material collected at the end of the experiment (TTO_2, 144h, **Figure 1**) was used for total microbial DNA extraction with the innuPREP Plant DNA Kit (Analytik Jena, GmbH, Germany). Cell lysis was performed with the SDS-based OPT lysis buffer (provided in the kit) and a chemical disruption was included by beating samples in a Precellys (6500 rpm 3 x 30 s), before continuing the extraction following the manufacturer’s instructions. DNA was quantified using a NanoDrop and the internal transcribed spacer 2 region (ITS2) was amplified with fITS7/ITS4 primers (GTGARTCATCGAATCTTTG/TCCTCCGCTTATTGATATGC) (Ihrmark et al., 2012). The dual indexing of amplicons was performed using Nextera XT index kit v2 and libraries were purified using the CleanNGS kit (CleanNA, Waddinxveen, Netherlands). Cleaned products were mixed in equal molar proportions and sequencing was conducted in a MiSeq Illumina sequencer at the Genotyping Service of the University of the Basque Country (SGIKER) using MiSeq Reagent Kit v2 (PE 2 x 250 bp, 500 cycles). In addition, 50 mL of each treatment (UL1, UL2, RU1 and RU2), stored at -80 °C the day they were applied, were thawed and centrifuged at 4000 rpm for 20 min. The pellets were used for DNA extraction, ITS2 library preparation and MiSeq sequencing following the aforementioned procedure.

Sequences were quality trimmed and demultiplexed in QIIME 2 (Bolyen et al., 2019). DADA2 was used for denoising, merging, chimera removing and amplicon sequence variant (ASV) determination. The leaves and algae extracts were taxonomically classified against the UNITE database (v8 04.02.2020). The alpha diversity of the leaves was determined based on Faith’s Phylogenetic Diversity index, while the Kruskal-Wallis test was used to test for significant differences in richness between treatments. Community composition differences based on Bray-Curtis index were visualized by principal coordinate analysis (PCOA) plots and significance was tested using the PERMANOVA test. The taxonomic composition of the leaf samples and algae extracts were inspected using bar plots. The taxa significantly differing in abundance between samples receiving algae extract and water treated samples were determined by linear discriminant analysis of effect size (Lefse) (https://huttenhower.sph.harvard.edu/galaxy/) setting the significance at a Kruskal-Wallis Bonferroni p value < 0.05, Wilcoxon test p < 0.01 and LDA > 2. In addition, differences between the mean relative abundance of particular beneficial groups known for their biocontrol activity were evaluated by the Mann-Whitney U test (significance threshold p < 0.05) using SPSS 28.0 (IBM Corp.).

## Results & discussion

### Characterization and biochemical composition of the extracts

The *Ulva ohnoi* extracts (UL1 and UL2) elaborated for this study contained lower total carbohydrate values (14-24 %) and a higher protein content in the case of UL1 (**Table 2**) than the data previously described for this species (Kidgell et al., 2019). These discrepancies are not surprising, as protein and carbohydrate are highly dependent on external conditions such as temperature, light intensity and nutrient concentration in water, and therefore vary with the season and habitat of collection (Hentati et al., 2020; Lafarga et al., 2020). Comparing both *Ulva* extracts showed that UL1 had a higher concentration of uronic acid and sulfate than UL2 (**Table 2**). This may suggest a higher bioactivity of UL1 regarding antimicrobial activity (Ibrahim et al., 2022) as uronic acid and sulfate groups of ulvan, the main polysaccharide found in *Ulva spp*, have previously been described to be bioactive (Guidara et al., 2021).

**Table 2:** Biochemical composition of *Ulva ohnoi* and *Rugulopteryx okamurae* extracts.

	Ash (%)	Carbohydrates (%)	Proteins (%)	Lipids (%)	Sulfate (%)	Uronic (%)	Fucose (%)	C (%)	H (%)	N (%)	S (%)	C/N
UL1	26.16 (0.74)	23.79 (0.04)	24.09 (1.38)	5.81 (0.41)	55.18 (0.14)	19.78 (1.52)	7.75 (0.50)	31.58	5.97	4.38	0.81	7.21
UL2	49.03 (0.84)	13.87 (0.01)	5.01 (1.49)	5.43 (0.33)	43.49 (0.03)	12.30 (1.09)	7.74 (0.28)	15.28	4.52	0.91	4.62	16.79
RU1	26.36 (1.43)	14.40 (0.04)	10.23 (1.55)	4.21 (0.26)	59.07 (0.11)	7.66 (2.85)	1.07 (0.20)	37.50	6.12	1.86	0.15	20.16
RU2	32.00 (0.64)	12.79 (0.02)	2.31 (0.21)	8.29 (0.79)	23.76 (0.07)	4.39 (1.00)	1.01 (0.08)	27.98	5.25	0.42	0.04	66.62

Results are referenced to extract dry weight and expressed in % as the means of samples analyzed in triplicate (n=3) except for CHNS. Standard deviation between brackets.

In contrast to *Ulva*, the composition of *Rugulopteryx okamurae* has been scarcely described. A recently published work by Cuevas et al. (2021) found 18.47 ± 0.35 % ashes, 9.76 ± 0.16 % proteins, and 11.63 ± 0.22 % lipids in lyophilized algae collected from the Cádiz coast (close to the studied area in the present work). In contrast, the RU1 and RU2 extracts studied here ranged between 26-32 % for ashes, 2-11 % for proteins, 4-9 % for lipids, and 12-15 % for carbohydrates. Sulfate and uronic acid content were higher in RU1 (59.07 % and 7.66 % respectively) than in RU2 (23.76 % and 4.39 % respectively), while fucose content was similar in both RU extracts. Importantly, RU2 exhibited a particularly high C/N ratio due to its low nitrogen content.

Macroelements, microelements, and heavy metals in the extracts were analyzed (**Supplementary Table 1**). Some data have previously been reported regarding the mineral composition in fresh algae (Maehre et al., 2014), but as far as we know, no data on the mineral composition of extracts have been published. UL1 was rich in Fe, Cr and Cu, while RU2 showed a high concentration of Na and As. Besides, the composition of the extracts complied with the legal requirements imposed by the EU to be used as fertilizers (https://eur-lex.europa.eu/legal-content/EN/TXT/?uri=CELEX%3A52016PC0157).

### Gene expression

Most seaweed extracts are able to elicit specific responses from the innate immunity of plants. The activation of signaling pathways leads to an increased expression of gene encoding: (i) pathogenesis-related (PR) proteins with antifungal and antibacterial activities; (ii) defense enzymes such as phenylalanine ammonia lyase (PAL) and lipoxygenase (LOX), which determine the accumulation of phenylpropanoid compounds and oxylipins with antiviral, antifungal and antibacterial activities; and iii) enzymes involved in the synthesis of terpenes, terpenoids and/or alkaloids (Vera et al., 2011; Bodin et al., 2020).

**Figure 2:**
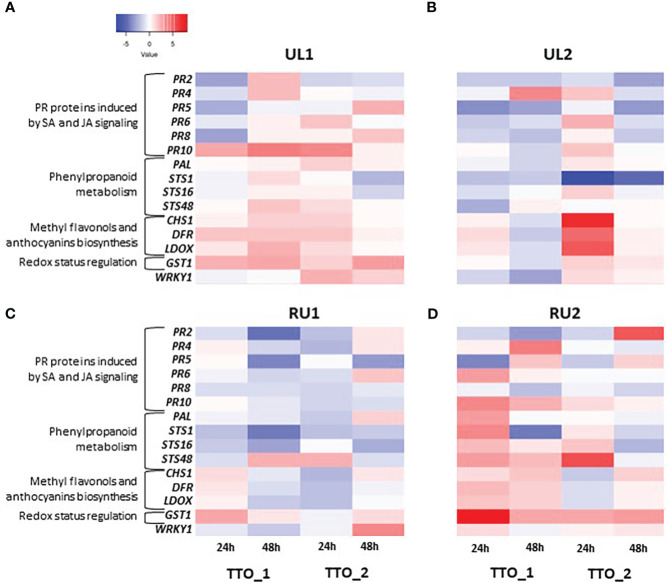
Transcript levels of defense-related genes in leaves induced by algae extract applications on grapevine plants **(A)** UL1; **(B)** UL2; **(C)** RU1; **(D)** RU2). Each column represents the sampling time, and each row represents one gene. A tree color scale was used to show fold induction of each gene (log transformed). The fold induction values were normalized to the reference genes PDC, GAPDH and COX and to water-treated leaves as the control samples.

The analysis of the Tempranillo greenhouse plants showed that the pool of *PR proteins* behaved differently according to the treatment and sampling point. The most induced protein was the Ribonuclease-like *PR10*, which has been shown to be induced by pathogen attack in a wide variety of plant species (Hashimoto et al., 2004), suggesting that grapevines could recognize the algae extract compounds as an elicitor of plant defense (Delaunois et al., 2014). *PR10* was upregulated by RU2 and UL1 24h and 48h after the first application (TTO_1), and only 24h after a second application (TTO_2) (**Figures 2A, D**). Previous studies performed in grapevine showed a long-lasting overexpression of PR10 protein after treatment with *Ascophyllum nodosum-*derived extract, where *PR10* was still induced up to two weeks post application (Bodin et al., 2020). Other PR proteins were either induced at 24h or at 48h after the application of the seaweed extracts. For instance, *PR6*, the main function of which is to inhibit proteolytic enzymes of fungal origin (Sels et al., 2008), was slightly upregulated 24h after the second application (TTO_2, 24h) by UL1 and UL2 (**Figures 2A, B**), while in RU2 its induction was detected 24h after first application (TTO_1, 24h) (**Figure 2D**). *PR4* and *PR2*, on the other hand, showed a later response. *PR4* proteins, classified as endochitinases able to bind chitin (Brunner et al., 1998), were upregulated at 48h TTO_1 by UL1, UL2 and RU2 (**Figures 2A, B, D**). Similarly, *PR2*, involved in the degradation of the cell wall of invading fungal pathogens (Leubner-Metzger and Meins, 1999), was highly upregulated by RU2 at 48h TTO_2 with a fold induction as high as 5.29 (**Figure 2D**). In addition, it was upregulated to a lesser extent by UL1 at 48h TTO_1 (**Figure 2A**). In accordance with our results, *PR2* has been shown to be induced by a sulfated laminarin elicitor in grapevine (Gauthier et al., 2014).

Expression of PR protein families is linked to hormonal signaling, being *PR10, PR4* and *PR6* induction mainly related to JA signaling, in contrast to *PR2* regulation that is known to be more related to SA signaling (van Loon et al., 2006; Guerreiro et al., 2016; Dermastia et al., 2021).

The *WRKY1* transcription factor was upregulated by UL1 24h after the second application (TTO_2, 24h, **Figure 2A**), and after 48h by RU1 (TTO_2, 48h, **Figure 2C**). This factor can correlate with *PR10* expression and participates in the oxidative burst induction and in H_2_O_2_ cellular accumulation (Guo et al., 2014).

Overall, the remaining pathways studied, including anthocyanins and flavonols accumulation, and biosynthetic pathways of phenylpropanoids and stilbene synthesis, were mostly induced 24h after the algae applications. For instance, UL2 treatment generated a 6.87-, 4.63- and 5.46-fold inductions for *CHS1, DFR* and *LDOX*, respectively (TTO_2, 24h, **Figure 2B**). Similar results were reported by Bodin et al. (2020), who found that two days after *A. nodosum* extract application *DFR* and *LDOX* genes were also up-regulated. The genes involved in the biosynthetic pathways of phenylpropanoids and stilbene synthesis were especially induced by RU2. *STS48* was particularly induced after two applications (TTO_2, 24h, **Figure 2D**), while *PAL* and *STS* proteins were mostly induced after the first treatment. *PAL* and *STS* overexpression correlated with *GST1* induction after *A. nodosum* extract treatment (Bodin et al., 2020). Similarly, *GST1* was highly upregulated at all sampling points by RU2 and to a lesser extent by UL1 (**Figures 2A, D**). GST proteins are involved in the detoxification of reactive molecules such as membrane lipid peroxides by conjugation to glutathione (Conn et al., 2008), in glutathione peroxidation to detoxify reactive oxygen species (Bartling et al., 1993), and in the transport and accumulation of phenylpropanoid compounds into the vacuole (Tavares et al., 2013). Importantly, both *PAL* and *STS* were reported to be major genes in the resistance against fungus of *Vitis vinifera* L. (Kortekamp, 2006). Similarly, the upregulation of *GST1* has been shown to correlate with stilbene-related genes in response to fungal infection (Gruau et al., 2015; De Bona et al., 2019). Therefore, the results from the present study suggest the potential in particular of the *Rugulopteryx okamurae*-derived extract RU2 as a biostimulant, triggering grapevine defenses at early stages.

### Phenolic compounds

In grapevine, polyphenols are present as constitutive compounds of the lignified organs (roots, canes, seeds, stems, ripe cluster stems) and/or as induced substances in leaves and berries. Among the phenolic families of compounds, the stilbenes are well-known phytoalexins counteracting oxidative stress and activating defense pathways (Gabaston et al., 2017; Šamec et al., 2021).

Up to 132 phenolic compounds, including 40 stilbenes, have been described in grapevine leaves (Goufo et al., 2020). Flavonols (mainly quercetins) are usually found in leaves. However, their detection depends on both the grapevine variety and extraction method (Fernandes et al., 2013). In the present work, flavonols were only detected in a few samples and were under the limit of quantification. Two flavonols (quercetin-3-*O*-glucurinide and an unknown flavonol) were detected in the UL2 leaf samples 24h after the second treatment (TTO_2, 24h), coinciding with the strong induction of *CHS1, DFR* and *LDOX* genes observed at this sampling point for the leaves treated with this extract (**Figure 2B**).

The main polyphenols detected here in grapevine leaves were those belonging to hydroxycinnamic acids, such as *trans*-caffeoyltartaric acid (*t*-caftaric), *trans*-coumaroyltartaric acid (*t*-coumaric), and *cis*-coumaroyltartaric acid (*c*-coumaric) (**Table 3**). *t*-Caftaric acid was predominant among this family of compounds, ranging from 676 mg/kg DW (RU2, TTO_1, 48h) to 2889 mg/kg DW (Water, TTO_2, 24h). These compounds are known to play a role in epidermal UV-screening, and therefore reflect light conditions, but they are not commonly found to be involved in stress response (Latouche et al., 2013). Overall, the measurements obtained in the present study were highly variable and dependent on the sampling date and treatment.

**Table 3:** Polyphenol content (mg/kg DW) in treated leaves at different sampling times.

TTO_1, 24h						
	Water	UL1	UL2	RU1	RU2	L.S
***t*** **-Caftaric acid**	1905.6 (493.4) a	1154.2 (596.2) b	2245.2 (567.7) a	1901.2 (197.8) a	2204.4 (383.1) a	***
***t-*** **Coutaric Isom**	65.97 (25.30)	49.66 (31.97)	53.87 (41.14)	58.75 (22.63)	82.43 (11.16)	n.s
***c-*** **Coutaric acid**	176.37 (34.22) ab	134.22 (48.15) ab	114.66 (88.55) b	151.75 (13.09) ab	198.27 (29.04) a	**
***t*** **-Piceid**	0.07 (0.00) b	1.16 (0.94) b	0.07 (0.00) b	0.07 (0.00) b	8.65 (6.52) a	***
***t*** **-Resveratrol**	0.07 (0.00) b	21.18 (10.38) abc	7.42 (2.53) bc	28.32 (22.68) ab	40.57 (27.00) a	***
TTO_1, 48h						
	**Water**	**UL1**	**UL2**	**RU1**	**RU2**	**L.S**
***t*** **-Caftaric acid**	1707.8 (337.5) a	938.8 (391.5) bc	1639.8 (869.5) ab	1067.5 (152.6) abc	676.4 (309.3) c	***
***t-*** **Coutaric Isom**	82.45 (14.47) ab	67.53 (16.68) ab	87.29 (25.74) a	72.94 (13.45) ab	57.19 (19.39) b	*
***c-*** **Coutaric acid**	169.06 (20.24) a	127.85 (31.87) ab	133.19 (41.14) ab	122.41 (18.98) ab	99.74 (36.57) b	**
***t*** **-Piceid**	4.77 (1.24)	8.25 (7.12)	4.98 (2.15)	5.36 (4.76)	5.60 (4.49)	n.s
***t*** **-Resveratrol**	16.58 (12.11)	12.05 (11.73)	10.77 (8.65)	25.04 (23.21)	10.03 (4.54)	n.s.
TTO_2, 24h						
	**Water**	**UL1**	**UL2**	**RU1**	**RU2**	**L.S**
***t*** **-Caftaric acid**	2889.1 (447.2) a	1861.8 (1165.0) b	2107.2 (919.5) b	1838.8 (282.6) b	1726.6 (341.6) b	*
***t-*** **Coutaric Isom**	84.08 (12.05)	73.64 (18.66)	71.54 (9.65)	70.63 (7.58)	67.88 (5.69)	n.s.
***c-*** **Coutaric acid**	221.55 (31.61) a	151.36 (46.63) b	162.78 (45.10) b	152.07 (16.18) b	147.96 (7.15) b	***
***t*** **-Piceid**	0.07 (0.00) c	10.42 (1.47) a	0.07 (0.00) c	0.07 (0.00) c	3.05 (0.65) b	***
***t*** **-Resveratrol**	19.06 (13.11) ab	35.26 (24.97) a	10.02 (7.37) b	29.44 (7.91) ab	27.42 (10.25) ab	*
TTO_2, 48h						
	**Water**	**UL1**	**UL2**	**RU1**	**RU2**	**L.S**
***t*** **-Caftaric acid**	1542.9 (645.5)	2416.0 (1082.0)	1921.1 (744.3)	1387.2 (192.4)	1587.4 (126.6)	ns
***t-*** **Coutaric Isom**	63.11 (9.82) b	88.42 (17.10) a	80.91 (14.63) ab	70.97 (11.66) ab	73.93 (7.57) ab	*
***c-*** **Coutaric acid**	141.83 (38.00) b	207.12 (56.25) a	180.77 (42.63) ab	141.54 (13.47) b	124.88 (50.78) b	**
***t*** **-Piceid**	0.07 (0.00) b	11.62 (5.41) a	1.93 (1.44) b	2.47 (1.48) b	3.01 (1.65) b	***
***t*** **-Resveratrol**	14.14 (7.77)	18.52 (12.65)	19.00 (16.39)	28.47 (15.42)	19.99 (12.55)	ns

Water: leaves treated with water (Control), UL1: leaves treated with UL1, UL2: leaves treated with UL2, RU1: leaves treated with RU1, RU2: leaves treated with RU2. Results are the means of three independent samples analyzed in triplicate. Standard deviations are indicated between brackets. Different letters for the same parameter denote significant differences (p < 0.05). Analysis of variance. Level of significance (LS): *p < 0.05, **p < 0.01, ***p < 0.001; ns: not significant.

Regarding stilbenes, significant differences in the concentration of *t*-piceid and *t*-resveratrol were observed between treatments, especially after the first application (TTO_1, 24h). At this sample point, the leaf samples treated with RU2 extract showed the highest stilbene values among the treatments (8.65 and 40.57 mg/kg DW leaf of *t*-piceid and *t*-resveratrol, respectively), which coincides with the observed activation of the defense mechanisms associated with phenylpropanoid genes for this extract (*PAL, STS1* and *STS48*; **Figure 2D**). Similar results have been described for a commercial algae extract applied at 5 g/L dosage tested as an elicitor on Marselan plants (Krzyzaniak et al., 2018), where *t*-piceid reached 10 mg/kg DW after 24 hours of the treatment. However, 48h after the first treatment (TTO_1, 48h), the concentrations of stilbenes in the RU2 samples decreased to values similar to those found in the control plants (**Table 3**). While these data agree with what was described for *t*-resveratrol elicitation after a plant extract application in Krzyzaniak et al. (2018), conversely these authors described that *t*-piceid concentration was maintained 48h after the treatment. Unfortunately, the authors did not describe the composition of the product applied, and therefore it is not possible to make further comparisons . When studying the plants receiving two applications, the increase in stilbenes was again evident mainly after 24h (TTO_2, 24h, **Table 3**), especially in the UL1 leaf samples. *t*-Piceid was strongly induced in UL1, while *t*-resveratrol increased in UL1, RU1 and RU2, but was significant only for UL1 with regard to the control (water). After 48h (TTO_2, 48h) only UL1 maintained a high *t*-piceid concentration (**Table 3**).

Therefore, the results suggest that RU2 and UL1 were the extracts with highest capacity for phytoalexin production, inducing stilbene biosynthesis, and therefore showing promising results as biostimulant products.

### Endogenous plant hormones

Phytohormones are key molecules involved in several processes throughout plant growth and development (Ross et al., 2011; De Diego et al., 2012). They play an essential role in the ability of plants to respond to different stress situations, either abiotic (e.g. drought) or biotic (e.g. pathogen attack), by mediating a wide range of adaptive responses (Santner and Estelle, 2009). The complexity of plants’ response includes hormone synthesis, transport and signaling pathways, and many interactions between them. In addition, hormone signaling and gene expression form a network in which relevant genes regulate hormone activities and *vice versa* (Liu et al., 2017). Gene expression results (Section 3.2) pointed to an early response of grapevine to algae amendment, and therefore in the present work the levels of the main phytohormones involved in plant growth regulation and plant defense mechanisms were studied in the samples from 24 hours after the first treatment (TTO_1, 24h).

**Figure 3:**
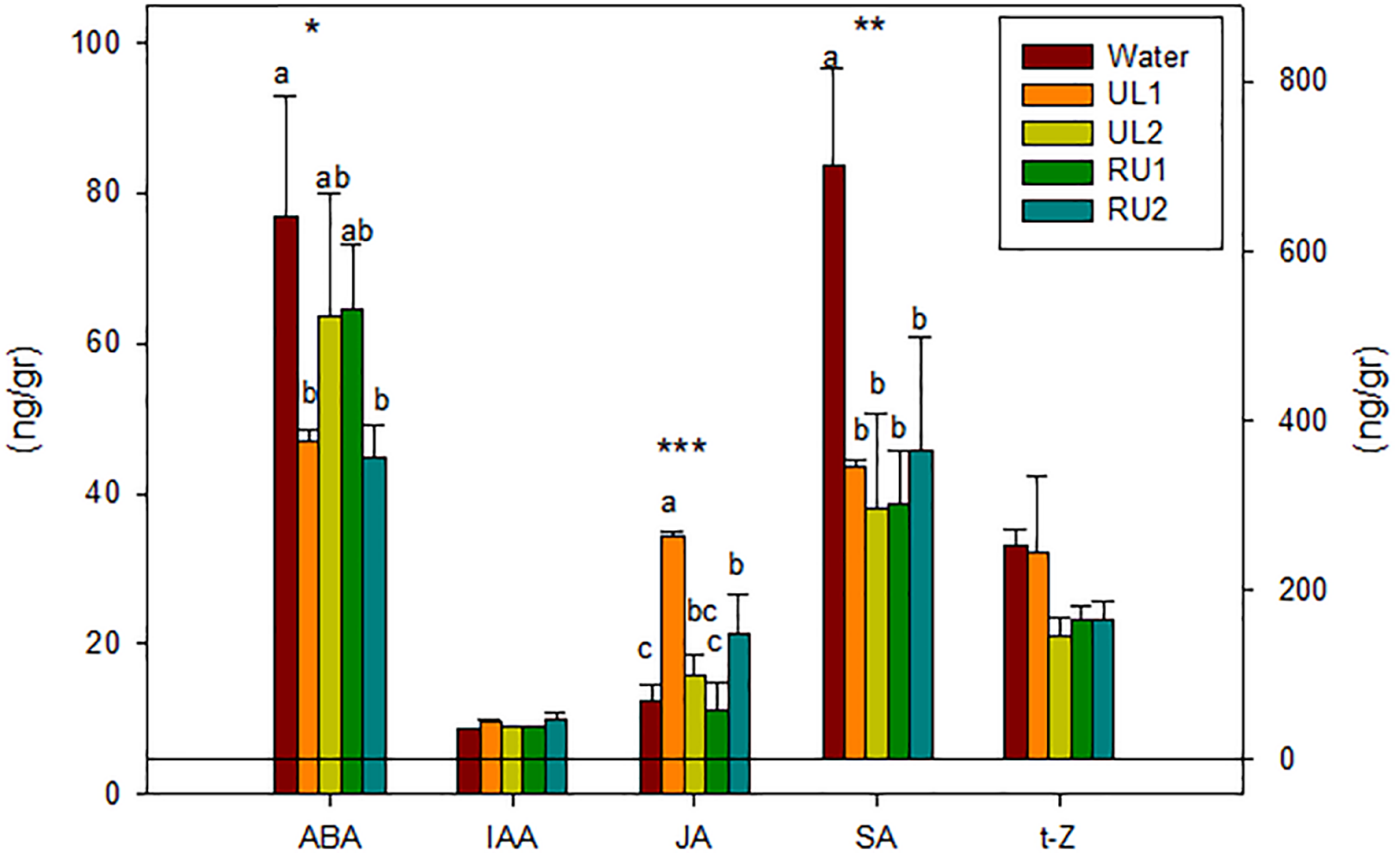
Leaves phytohormones content 24 hours after first treatment (TTO_1, 24h) (ng/g DW). ABA, Abscisic acid; IAA, Indolacetic acid; JA, Jasmonic acid; SA, Salycilic acid; t-Z, trans-zeatine. Analyses of variance. levels of significance (LS): *p < 0.05, **p < 0.01, ***p < 0.001.

The results show that the extracts significantly reduced the total content of hormones, this reduction ranging between 40 % in UL1 and RU2, and 50 % in UL2 and in RU1 treated plants, in comparison with the water-treated samples (**Figure 3**). SA was the most abundant hormone, constituting 67 % of total hormone content found in the control plants and being the one that showed the highest decrease (by 50 % in the treated plants). The *t*-Z, which is considered one of the most active CK forms (Lacuesta et al., 2018), was the only CK detected in the Tempranillo leaf samples, iP and ZR being under the detection limit. Neither the *t*-Z levels nor the auxin IAA were significantly affected by the algae extract treatments (**Figure 3**).

JA was the only hormone to increase its content with the algae treatments when compared to water, especially in the UL1 and RU2 leaves. These plants were also the ones that showed the greatest decrease in ABA (**Figure 3**). These results reinforce the previously reported findings that ABA is an important signal in the activation of plant defenses by a possible enhancement of JA synthesis (Adie et al., 2007; Trotel-Aziz et al., 2019).

JA has been reported to enhance the tolerance of grapevine foliar cuttings and vineyards to the pathogen *Eryshiphe necator* in cv. Cabernet Sauvignon and has been associated with an increase in transcript levels of PR proteins, phytoalexin biosynthesis, and with the accumulation of stilbenes (Belhadi et al., 2006). Similar to the mentioned studied, JA induction corresponded with the up-regulation of *PR10* (in RU2 and UL1) and *PR6* (in RU2) (**Figure 2D**) 24 hours after the first application (TTO_1, 24h), as well as with the significant accumulation of stilbenes in the UL1 and RU2 samples (**Table 3**). In the same line, Cruz et al. (2019) evidenced that external applications of JA and methyl jasmonate (a derivative of JA) are potent elicitors that act as signaling molecules upon biotic stress and are involved in plant defense mechanisms leading to an amplified endogenous jasmonate response (Paolacci et al., 2017) as can also be seen in **Figure 3**, or triggering the synthesis of secondary compounds such as stilbenes (**Table 3**).

In contrast to the JA induction, SA content after the first application (TTO_1, 24h) decreased for all the algae treated samples compared to water. The studied Tempranillo plants seemed to be activating the SA-dependent defense response later in the experiment, as the SA induced gene *PR2* was found to be overexpressed at (TTO_1, 48h). Thus our results suggest that the JA signaling is negatively regulating the expression of SA-mediated defenses. The antagonistic activity of JA and SA signaling found in the present study after algae extract amendment has been previously evidenced by other authors after elicitors in grapevine and other species (Gupta et al., 2000; Kunkel and Brooks, 2002; Paolacci et al., 2017; Trotel-Aziz et al., 2019).

Overall, our results suggest that the seaweed extracts are mainly activating the defense mechanisms of grapevines through JA synthesis, RU2 and UL1 being the extracts that showed a higher hormonal response.

### Enzymes related to plant defense

Increasing the activity of enzymes within the antioxidant defense system of the plant is considered to be an effective mechanism to combat the oxidative stress induced by various stresses (e.g. drought, chilling, UV irradiation, exposure to intense light, wounding and pathogens) (Sharma et al., 2012 ). In the present work, the main antioxidant enzymes were measured, including GR, SOD, CAT and APX enzymes. These enzymes are known to be involved in scavenging the toxic ROS (reactive oxygen species). ROS drastically increase in plants in response to environmental stresses, being a main player in plant growth and the improvement of plant tolerance to stress (Das and Roychoudhury, 2014).

**Figure 4:**
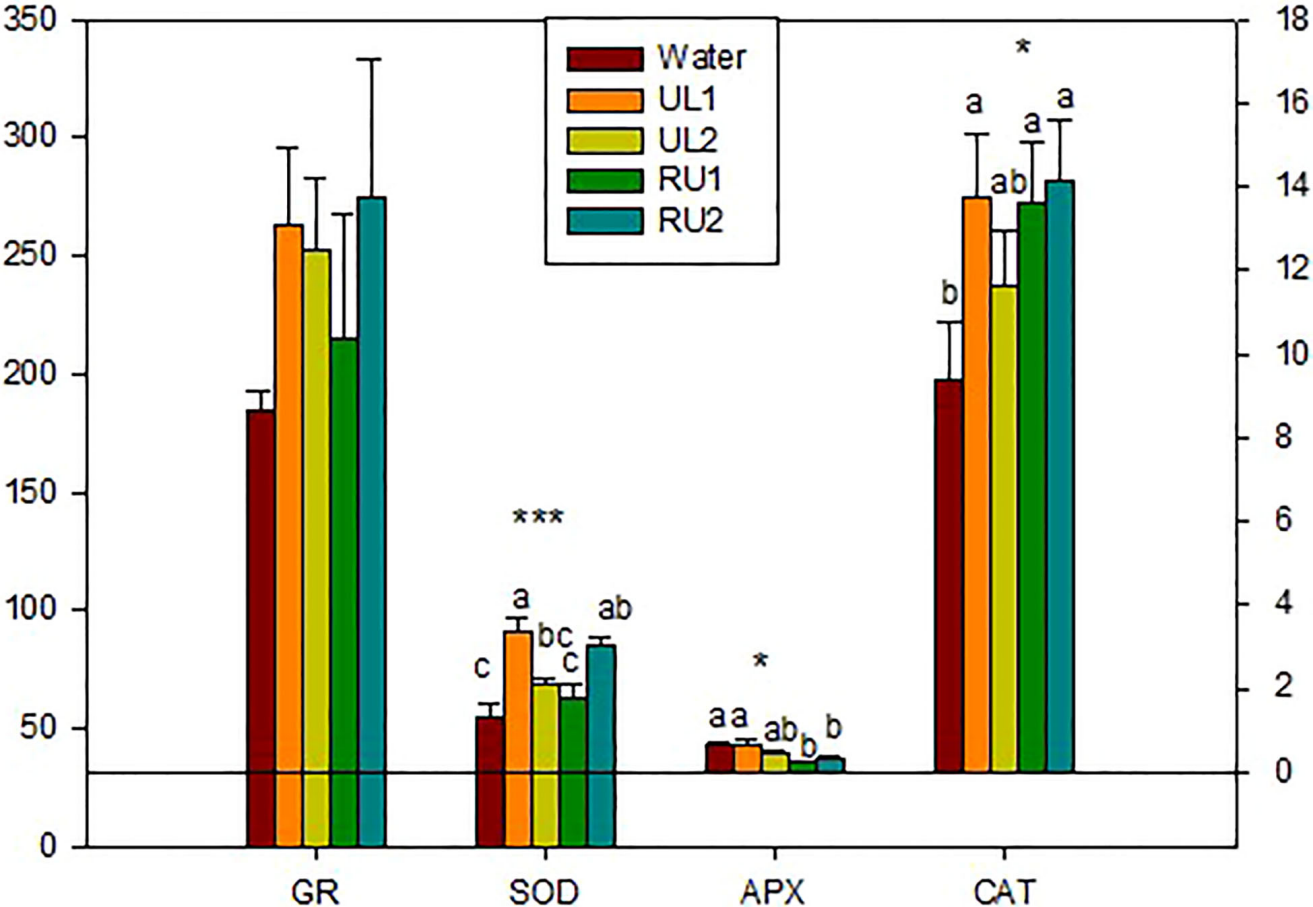
Enzymes related to plant defense at the end of the experiment. SOD, superoxide dismutase (U/mg protein); APX, ascorbate peroxidase (mmol Ascorbate/min mg protein); CAT, catalase (nmol H2O2/min mg protein); GR, gluthatione reductase (nmol NADPH/min mg protein). Analyses of variance. levels of significance (LS): *p < 0.05, ***p < 0.001.

*GR* activity was not significantly modified by the treatments, but the lowest amounts were found in the CT samples (water) (**Figure 4**). A significant increase in SOD (p < 0.001) and CAT (p < 0.05) was observed in UL1, RU1 and RU2. However, APX significantly decreased in RU1 and RU2 (p < 0.05) (**Figure 4**). As it is known, SOD catalyzes the dismutation of the superoxide radical, generating H_2_O_2_ which is then eliminated by both APX and CAT, among other enzymes. The contrasting values observed in both RU1 and RU2 between SOD (increasing) and APX (decreasing) might suggest that they are promoting the accumulation of H_2_O_2_. Importantly, H_2_O_2_ has been shown to inhibit pathogens directly or by generating other free radicals with antimicrobial activity that could also be toxic for fungi (Raj et al., 2018). Liu et al. (2015) demonstrated that *Vitis vinifera* genotypes resistant to *Plasmopara viticola* accumulated H_2_O_2_, while the susceptible *Vitis vinifera* Pinot noir did not. Other authors have described an induction of SOD in grapevine leaves after infection with this fungus (Wang et al., 2022). A potential accumulation of H_2_O_2_ seems contradictory with the increase of CAT, since catalase detoxifies H_2_O_2._ However, the reason for this discrepancy could be that CAT levels increase to eliminate the H_2_O_2_ produced due to higher photorespiration. At this point we cannot elucidate which are the specific molecules (polysaccharides, lipids, metals, among others) detected by the receptors of the host plant to induce the observed response. A direct measurement of the potential H_2_O_2_ accumulation in RU2 by chemiluminescence assay would be necessary to confirm the explanations raised.

### Vine development and photosynthetic pigments

Many biostimulants are thought to enhance nutrition efficiency, biotic stress tolerance, crop yield, plant physiology (Sabir et al., 2014; Salvi et al., 2019) and plant growth (du Jardin, 2015; Yakhin et al., 2017). The analysis of the content of photosynthetic pigments is a direct indicator of the plant’s ability to respond adequately to biotic stress since they are essential to synthesize the carbohydrates necessary for both plant growth and to serve as carbon skeletons for the different pathways of secondary metabolism.

The seaweed extracts applied in the present study, however, did not show a significant effect on i) growth parameters (the leaf number/plant, stem height and root system biomass), ii) the composition of photosynthetic pigments (chlorophylls and carotenoids), or iii) the maximum photochemical efficiency in the light of photosystem II (*φ_PSII_*), at the end of the experiment (TTO_2, 144h, **Supplementary Table 2**). These data suggest that the treatments performed were not efficient as biofertilizers, in agreement with the CK hormone data (**Figure 3**). Treating for longer periods or performing more intensive treatments might be necessary to observe changes in the morphophysiological characteristics of grapevines.

### Leaf fungal community diversity and composition

Leaf-associated microorganisms are involved in the diffusion of xenobiotics and represent a barrier against pathogens by activating plant defenses and competing with pathogenic organisms (Bulgarelli et al., 2013; Trouvelot et al., 2014). The stimulation of the beneficial microbiota of the grapevine phyllosphere by protein-derived products, carbohydrate-based treatments and commercial elicitors has been previously evidenced in grapevines cultivated in greenhouses (Cappelletti et al., 2016) and field experiments (Perazzolli et al., 2014; Nerva et al., 2019). This contributes to plant health by enhancing biocontrol activities against phytopathogens (Perazzolli et al., 2020). Seaweed extracts are therefore expected to alter the abundance of particular taxa in grapevine leaves, which could play an indirect role in their protection.

**Figure 5:**
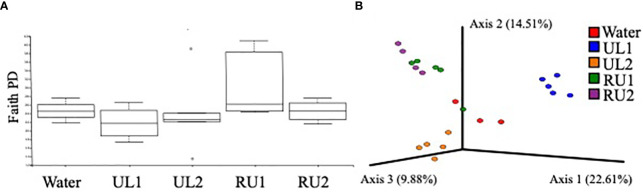
**(A)** Alpha diversity plot showing the mean fungal phylogenetic diversity by treatment. No significant differences in Faith PD index were observed between treatments (Kruskal-Wallis H= 4.710, p= 0.318). **(B)** PCOA plot showing fungal community composition dissimilarity between treatments (based on Bray-Curtis distance) at the end of the experiment (144h after the second application). Fungi composition significantly differed between treatments (PERMANOVA Pseudo-F= 3.927, p= 0.001). ASV tables were rarified to 20000 sequences per sample, so samples that did not reach that sequencing depth were excluded from the analysis.

The results from the present study evidenced that the foliar treatments with algae extracts did not significantly alter the fungal phylogenetic diversity of grapevine leaves (Kruskal-Wallis H= 4.710, p-value= 0.318), although the RU1 samples showed the highest variability (**Figure 5A**). However, the fungal composition of the leaves (based on Bray-Curtis index) differed significantly between treatments (PERMANOVA pseudo-F= 3.927, p-value= 0.001). The samples associated with each of the treatments were grouped into separated clusters in the PCOA plot (**Figure 5B**). UL1 mycobiota composition differed the most from the water-treated samples. In agreement with this, LefSe analysis, comparing the relative abundances of fungal groups of the samples receiving the algae treatments with leaf samples receiving water (**Supplementary Table 3**), identified the highest number of biomarkers for UL1, which showed a significantly higher abundance of Sporidiobolaceae (genera *Apiotrichum* and *Rhodotorula)*, an anti-phytopathogenic microorganism known to be common in grapes and leaves (Pinto et al., 2014), as well as members of Filobasidiaceace (*Filobasidum magnum* and *Naganishia albida*) and Rhynchogastremaceae (*Papiliotrema albida*) (**Supplementary Table 3**). However, the source of these groups is likely the algae phylloplane, as these were the most abundant families encountered when sequencing this algae extract (**Supplementary Figure 1**). The study of microbial consortia in seaweed has attracted considerable attention lately as a result of the capacity of seaweed endosymbiotic microorganisms to generate antimicrobial and antioxidant compounds, as well as other molecules with biotechnological applications (Ren et al., 2022). Although the characterization of *Ulva ohnoi* and *Rugulopteryx okamurae* macroalgae microbial consortia was beyond the scope of this study, the sequencing of the extracts was performed. Aside from the above-mentioned genera present in UL1, Saccharomycetes were abundant in the extracts (particularly in UL2, **Supplementary Figure 1**). Importantly, the UL2 and RU2 Tempranillo leaf samples had an enrichment of *S. cerevisiae* compared to the control samples (**Supplementary Table 2**), with mean relative abundances of 18 % in UL2, 13 % in RU2 and 2 % in the water-treated samples (**Table 4**). Saccharomyces are part of the native microflora of grapevine leaves (Pinto et al., 2014). Therefore, aside from the accumulation of putative cells coming from the algae extract, the enrichment of *S. cerevisiae* in the leaf samples could in part be due to the response of the indigenous microbiota of the leaves to the treatments. While we cannot confirm *S. cerevisiae* activity, as amplicon sequencing method does not allow dead and active organisms to be discerned, studies conducted by (Mishko and Lutsky, 2020) evidenced that a pre-treatment of grape leaves with *S. cerevisiae* enhanced the immune response of a *Plasmopara viticola* resistant vine cultivar, while it induced phytoalexin synthesis (stilbenes) in a susceptible variety prior to the disease infection.

**Table 4:** Mean relative abundance (%) and standard deviation of beneficial genera known to have antifungal or antagonistic activity.

	*Trichoderma*	*Aspergillus*	*Penicillium*	*Fusarium*	*Aureobasidium*	*Candida*	*Rhodotorula*	*Debaryomyces*	*Sporobolomyces*	*Saccharomyces*
**Water**	0.04 (0.06)	0.12 (0.04)	0.56 (0.46)	0.14 (0.24)	0.84 (0.96)	1.11 (1.93)	0.09 (0.16) a	0.00 (0.00) ab	0.62 (1.07) a	2.26 (1.97) a
**UL1**	0.00 (0.00)	0.11 (0.11)	0.19 (0.07)	0.00 (0.00)	0.24 (0.41)	0.52 (1.02)	8.09 (1.07) b	0.00 (0.00) a	0.62 (0.47) a	0.35 (0.45) a
**UL2**	0.02 (0.05)	0.12 (0.13)	0.70 (0.33)	0.00 (0.00)	0.18 (0.34)	1.72 (1.81)	0.97 (0.82) a	0.68 (0.69) ab	0.82 (0.78) a	18.71 (4.72) b
**RU1**	0.00 (0.00)	0.13 (0.15)	0.52 (0.39)	0.06 (0.08)	0.40 (0.30)	0.60 (0.58)	0.60 (0.43) a	0.00 (0.00) a	1.03 (0.59) a	6.21 (5.51) ac
**RU2**	0.03 (0.05)	0.05 (0.04)	0.37 (0.14)	0.00 (0.00)	0.75 (0.87)	0.87 (0.96)	0.26 (0.30) a	2.32 (2.28) b	2.80 (1.29) b	13.03 (6.61) bc

The different letters indicate the between treatments significance according to Mann-Whitney U 2-sided test.

The seaweed treatments did not result in a clear shift in the abundance of various known biocontrol agents, such as *Trichoderma* (Pollard-Flamand et al., 2022), *Aureobasidium pullulans* (Harm et al., 2011), *Candida* (Sipiczki, 2006), among others (**Table 4**). These organisms were found in general at low abundances in the analyzed samples and showed a high variability within treatment plant replicates. However, other groups such as Sporobolomyces, known for their antifungal activity (Sipiczki, 2006), were particularly abundant in the RU2 samples compared with the water-treated leaves (**Table 4**). It was also noteworthy that *Debaryomyces hansenii* was significantly enriched in UL2 (0.68 % relative abundance), and particularly in RU2 leaves (2 %), while it was almost absent in the remaining treatments. Several strains within this genus exhibit antagonistic activity against fungal phytopathogens through diverse mechanisms, such as competition for nutrients and space, mycoparasitism, the secretion of antifungal substances (e.g. volatile organic compounds, glucanases, and killer toxins) or the induction of plants’ immune response to pathogens (Fernandez-San Millan et al., 2020).

Further analysis is needed to confirm the functional activity and strain classification of the microbial changes induced by the treatments and their role in grapevine protection, but the results highlight that fungal composition is partially altered with the enrichment of particular beneficial species, predominantly after RU2 foliar applications.

## Conclusion

In the current work, two crude extracts each from *Ulva ohnoi* and *Rugulopteryx okamurae* have been developed and biochemically characterized. The sulfate, uronic acid, fucose and metal content of *Rugulopteryx okamurae* have been described for the first time. However, a deeper characterization of the extracts’ carbohydrates (laminarin, fucoidan, alginates) and lipids (fucoxantine, fucosterol, glucolpids, terpenes and lipids from betaine) is required to establish a relationship between the seaweed composition and extract bioactivity.

The greenhouse assay conducted here on the Tempranillo variety suggests that the aqueous *Rugulopteryx okamurae* extract (RU2) induces grapevine defense and immunity genes, as well as secondary metabolites such as stilbenes, phytohormones and antioxidant enzymes involved in the protection and resistance to biotic/abiotic stress. In addition, RU2 enriched the abundance of fungal antagonists in the leaves. Further studies are needed to confirm the enhanced protection of the plants receiving RU2 extract, by evaluating the changes induced under pathogen inoculation. In addition, while the development of the vines was not altered by the algae extracts, further understanding of dosage and application spans, as well as the possible risks or benefits of RU2 for grapevine yield and grape quality would be necessary for its consideration as an alternative product with advantages for viticulture.

Evidencing *Rugulopteryx okamurae* efficacy as a biostimulator is a major finding as it would be a first step towards its inclusion in a circular scheme, reducing its accumulation in the coast and at the same time benefiting the viticulture sector.

## Data availability statement

The data (raw sequences) presented in the study are deposited in Qiita database repository under study ID-1024 (https://qiita.ucsd.edu/study/description/1024) and in ENA database with accession number ERP143695 (https://www.ebi.ac.uk/ena/browser/view/PRJEB58628).

## Author contributions

IZ, AD-N, AM-P, UP-L, ML and EP-A performed experiments (plants culture, development monitoring and sampling). CF-D and EC-V harvested/pick up the seaweed, performed the extracts and analyzed them. EC and NB performed analysis of gene expression. BP and EC-V developed the analysis of polyphenols. ML performed the analysis of hormones. UP-L performed the analysis of antioxidant enzymes. AM-P performed the vine development and photosynthetic pigments measurement. IZ performed the fungal community study. EC-V, IZ, AD-N, EC, AM-P, UP-L, ML and EP-A contributed to the experimental design. EC-V, IZ, EC, AM-P, UP-L and ML contributed to data interpretation. IZ and EC-V contributed to draft the manuscript and drafted led the project. All authors contributed to the article and approved the submitted version.
