# Supplementary material for: Holistic understanding of the response of grapevines to foliar application of seaweed extracts
Source: Front Plant Sci. 2023 Feb 24;14:1119854. doi: 10.3389/fpls.2023.1119854 (PMC10010106; doi:10.3389/fpls.2023.1119854)
Supplement: Supplementary file 1 [file DataSheet_1.pdf]

frontiers

Supplementary Material

Holistic understanding of the response of grapevines to seaweed extracts foliar application

Iratxe Zarraonaindia1,2*, Enrico Cretazzo3, Amaia Mena-Petite4, Ana Diez5, Usue Pérez-López6, Maite Lacuesta4, Eva Pilar Perez-Alvarez7, Belén Puertas3, Catalina Fernandez-Diaz8, Nadia Bertazzon9, Emma Cantos-Villar3*

*** Correspondence:** Corresponding Author: iratxe.zarraonaindia@ehu.eus; emma.cantos@juntadeandalucia.es

1 Supplementary Data

Supplementary Material should be uploaded separately on submission. Please include any supplementary data, figures and/or tables.

Supplementary material is not typeset so please ensure that all information is clearly presented, the appropriate caption is included in the file and not in the manuscript, and that the style conforms to the rest of the article.

2 Supplementary Figures and Tables

For more information on Supplementary Material and for details on the different file types accepted, please see here.

2.1 Supplementary Figures

Supplementary Figure 1. Relative abundance of most dominant fungal families (>1% abundance) identified in algae extracts and leaf samples (treated with water or algae extract) at the end of the experiment.

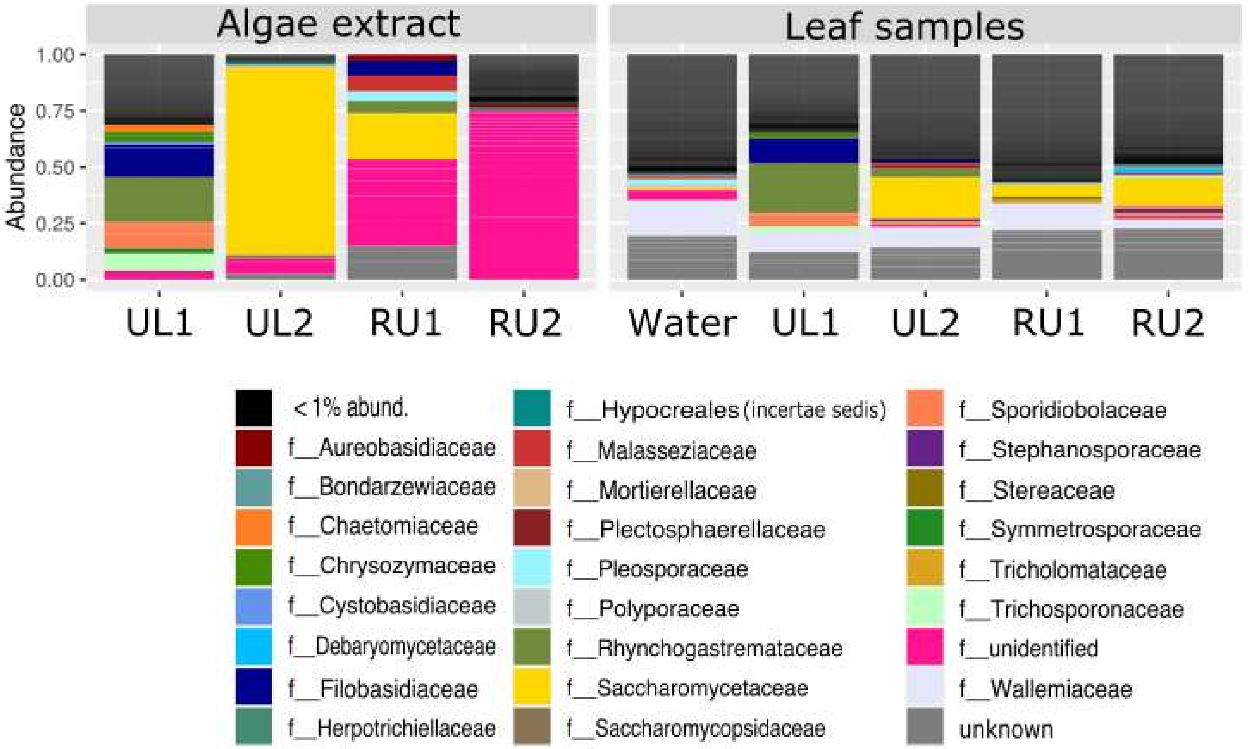

2.2 Supplementary Tables

Supplementary Table 1. Mineral composition of *Ulva ohnoi* and *Rugulopteryx okamurae* extracts (µg/g extract).

	Macroelements				Microelements						Heavy Metals			
	**Ca**	**K**	**Mg**	**Na**	**Fe**	**Mn**	**Cr**	**Cu**	**Zn**	**Se**	**Cd**	**Hg**	**Pb**	**As**
**UL1**	788.75	12591.62	25578.38	6440.40	1439.35	903.87	2.670	28.50	49.71	1.232	0.079	0.617	2.177	4.944
**UL2**	1680.34	40455.87	78124.68	18190.11	62.19	1273.29	0.267	1.24	7.19	0.756	0.027	0.617	0.131	5.283
**RU1**	4045.23	34049.72	6541.29	12071.26	265.33	17.03	0.889	2.76	24.22	1.507	0.149	0.664	1.412	33.380
**RU2**	2424.28	84885.94	10351.86	33692.55	12.62	11.03	0.178	0.29	8.36	1.009	0.017	0.641	0.336	79.970

Supplementary Table 2. Grapevines physiological parameters and pigments at the end of the experiment.

Physiological parameters	Water	UL1	UL2	RU1	RU2	LS
Chlorophyll a content (mg/g DW)	6.48 (0.45)	7.4 (0.63)	4.95 (0.56)	7.17 (0.87)	5.67 (0.94)	ns
Chlorophyll b content (mg/g DW)	2.56 (0.15)	3.19 (0.29)	2.17 (0.23)	3.08 (0.34)	2.37 (0.36)	ns
Carotenoids content (mg/g DW)	1.16 (0.16)	1.41 (0.09)	1.03 (0.10)	1.39 (0.13)	1.59 (0.23)	ns
Total chlorophylls (mg/g DW)	10.21 (1.31)	10.59 (0.91)	7.12 (0.78)	10.25 (1.21)	8.03 (1.3)	ns
Chlorophyll a/b ratio	2.11 (0.41)	2.32 (0.06)	2.28 (0.06)	2.32 (0.04)	1.83 (0.3)	ns
ϕ_{PSII}	0.71 (0.01)	0.736 (0.019)	0.714 (0.007)	0.724 (0.012)	0.726 (0.014)	ns
Greenness (SPAD values)	25.76 (0.79)	28.64 (1.07)	25 (1.72)	26.64 (2.15)	24.28 (1.19)	ns
Root dry weight (g)	3.82 (1.33)	4.42 (0.45)	4.04 (0.61)	2.74 (0.59)	3.76 (0.74)	ns
Root FW/DW	5.24 (0.47)	4.38(0.1)	4.39 (0.35)	4.79 (0.27)	4.72 (0.28)	ns
Leaf number/plant	13.80 (2.52)	18.00 (1.22)	15.60 (1.47)	13.40 (0.87)	16.20 (1.2)	ns
Stem height (cm)	82.40 (3.84)	98.40 (12.07)	69.00 (6.14)	86.00 (7.46)	82.60 (9.01)	ns

ϕ_{PSII} :maximum photochemical efficiency in the light of photosystem II; DW, dry weight; FW, fresh weight. Water: leaves treated with water (Control), UL1: leaves treated with UL1, UL2: leaves treated with UL2, RU1: leaves treated with RU1, RU2: leaves treated with RU2. Results are the means of three independent samples analyzed in triplicate. Standard deviations between brackets. Ns, no significance as a result of one-way analysis of variance (ANOVA)

Supplementary Table 3. Significantly enriched taxonomic groups with respect to water treated samples according to linear discriminant analysis effect size (LEfSe). The significance is based on Kruskal-Wallis Bonferroni p value <0.05, Wilcoxon test p<0.01 and LDA score (log10) >2. Horizontal bars represent the effect size for each taxon that showed a significant result. The length of the bar represents the log10 transformed LDA score.

Treatment	Phylum	Class	Order	Family	Genera	Species
UL1	Ascomycota	Dothideomycetes	Pleosporales	Didymellaceae		
	Basidiomycota					
		Cystobasidiomycetes	Incertae_sedis	Symmetrosporaceae	Symmetrospora	—
						foliicola
		Microbotryomycetes		Chrysozymaceae	Sampaiozyma	*vanillica*
			Sporidiobolales	Sporidiobolaceae	Rhodotorula	*diobovata*
						mucilaginosa
		Tremellomycetes	Filobasidiales	Filobasidiaceae	Filobasidium	*magnum*
					Naganishia	*albida*
			Tremellales	Rhynchogastremataceae	Papiliotrema	*frias*
			Trichosporonales	Trichosporonaceae	Apiotrichum	—
UL2	Ascomycota					
		Saccharomycetes	Saccharomycetales	Saccharomycetaceae	Saccharomyces	*cerevisiae*
	Basidiomycota	Agaricomycetes	Polyporales	Fomitopsidaceae	Fomitopsis	*pinicola*
		Tremellomycetes	Filobasidiales	Filobasidiaceae	Filobasidium	
			Tremellales			
RU1	Ascomycota	Dothideomycetes	Pleosporales	—	—	—
				Didymellaceae	Phoma	unidentified
	Basidiomycota	Agaricomycetes	Agaricales	Tricholomataceae	Clitocybe	*nebularis*
				Typhulaceae	Typhula	
			Auriculariales			
			Cantharellales	Incertae_sedis	Burgoa	unidentified
			Hymenochaetales	Schizoporaceae	Xylodon	*sambuci*
		Exobasidiomycetes	Entylomatales	unidentified	unidentified	unidentified
RU2	Ascomycota					
		Dothideomycetes	Pleosporales	Didymellaceae	Phoma	unidentified
				Pleosporaceae	—	—
		Saccharomycetes	Saccharomycetales	Debaryomycetaceae	Debaryomyces	
				Saccharomycetaceae	Saccharomyces	*cerevisiae*
	Basidiomycota	Agaricomycetes	Russulales			

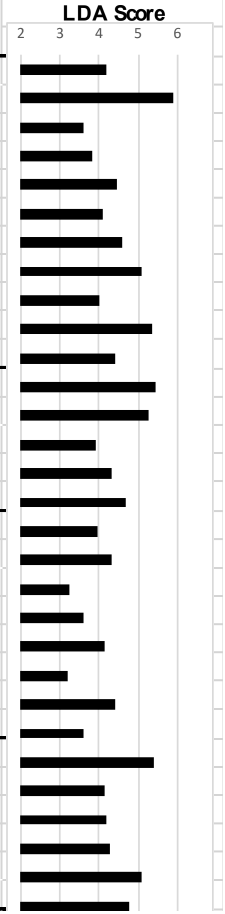

Water: leaves treated with water (Control), UL1: leaves treated with UL1, UL2: leaves treated with UL2, RU1: leaves treated with RU1, RU2: leaves treated with RU2.
